# Functional electrical stimulation therapy controlled by a P300-based brain–computer interface, as a therapeutic alternative for upper limb motor function recovery in chronic post-stroke patients. A non-randomized pilot study

**DOI:** 10.3389/fneur.2023.1221160

**Published:** 2023-08-17

**Authors:** Ana G. Ramirez-Nava, Jorge A. Mercado-Gutierrez, Jimena Quinzaños-Fresnedo, Cinthya Toledo-Peral, Gabriel Vega-Martinez, Mario Ibrahin Gutierrez, María del Refugio Pacheco-Gallegos, Claudia Hernández-Arenas, Josefina Gutiérrez-Martínez

**Affiliations:** ^1^Neurological Rehabilitation Division, Instituto Nacional de Rehabilitación “Luis Guillermo Ibarra Ibarra”, Tlalpan, Mexico; ^2^Medical Engineering Research Division, Instituto Nacional de Rehabilitación “Luis Guillermo Ibarra Ibarra”, Tlalpan, Mexico; ^3^Consejo Nacional de Humanidades, Ciencias y Tecnologías - Instituto Nacional de Rehabilitación “Luis Guillermo Ibarra Ibarra”, Tlalpan, Mexico

**Keywords:** Functional Electrical Stimulation Therapy, Brain-Computer Interface, P300 Event-Related Potential, stroke rehabilitation, upper extremity paresis, outcome assessment, activity-based therapy

## Abstract

**Introduction:**

Up to 80% of post-stroke patients present upper-limb motor impairment (ULMI), causing functional limitations in daily activities and loss of independence. UMLI is seldom fully recovered after stroke when using conventional therapeutic approaches. Functional Electrical Stimulation Therapy (FEST) controlled by Brain–Computer Interface (BCI) is an alternative that may induce neuroplastic changes, even in chronic post-stroke patients. The purpose of this work was to evaluate the effects of a P300-based BCI-controlled FEST intervention, for ULMI recovery of chronic post-stroke patients.

**Methods:**

A non-randomized pilot study was conducted, including 14 patients divided into 2 groups: BCI-FEST, and Conventional Therapy. Assessments of Upper limb functionality with Action Research Arm Test (ARAT), performance impairment with Fugl–Meyer assessment (FMA), Functional Independence Measure (FIM) and spasticity through Modified Ashworth Scale (MAS) were performed at baseline and after carrying out 20 therapy sessions, and the obtained scores compared using Chi square and Mann–Whitney *U* statistical tests (𝛼 = 0.05).

**Results:**

After training, we found statistically significant differences between groups for FMA (*p* = 0.012), ARAT (*p* < 0.001), and FIM (*p* = 0.025) scales.

**Discussion:**

It has been shown that FEST controlled by a P300-based BCI, may be more effective than conventional therapy to improve ULMI after stroke, regardless of chronicity.

**Conclusion:**

The results of the proposed BCI-FEST intervention are promising, even for the most chronic post-stroke patients often relegated from novel interventions, whose expected recovery with conventional therapy is very low. It is necessary to carry out a randomized controlled trial in the future with a larger sample of patients.

## 1. Introduction

Stroke is the second cause of death and a leading cause of long-term disability in adults globally (1). In Mexico, it is the fifth cause of mortality and the main cause of disability in adults. Although stroke mortality is decreasing, most survivors have long term sequelae (2). About 80% of post-stroke patients develop motor impairment commonly affecting one side of the body. Upper limb motor impairment (ULMI) affects 50–80% of individuals in the acute phase post-stroke and 40–50% in the sub-acute phase, causing functional limitations in activities of daily living and loss of independence (3–5).

Physical and Occupational Therapy are the main approaches of conventional stroke rehabilitation for ULMI recovery. Although the first one is based mainly on joint mobilization, muscular stretching and strengthening, both are focused on task specific training, functional tasks practice, constraint-induced movement therapy and activities of daily living (6–9). Despite all efforts, upper limb mobility is not always fully recovered after stroke (8, 10, 11). This suggests the need for more and better innovative technologies for rehabilitation of these cerebrovascular diseases. Among the wide variety of technologies that have been studied to assess their effects in the ULMI rehabilitation, we can mention electromyography-based biofeedback, virtual reality, electromechanical and robotic devices, transcranial magnetic stimulation, brain–computer interfaces (BCI), and functional electrical stimulation (FES) used as Functional Electrical Stimulation Therapy (FEST); nevertheless, these technologies are not widely available, and they are still in constant development and study (12, 13).

Recent studies have highlighted the use of electroencephalogram-based brain–computer interfaces (BCI) as a promising neurorehabilitation therapy that provides insight into the sensorimotor processes underlying motor function and motor learning after stroke (14). BCI systems may help to induce or facilitate neuroplasticity by strengthening connections between damaged areas of the nervous system, and to create a demand for reorganization of neuronal network functions (15).

On the other hand, some types of neural stimulation techniques have demonstrated benefits for ULMI recovery, one of the most promising being the combination of FES with BCI, since they may be used as an effective alternative to carry out task-oriented training as a motor rehabilitation strategy after stroke (16), and induce neuroplastic changes by directly linking movement intent with muscle contraction (17). Unfortunately, few studies have been done that evaluate this combination in controlled clinical trials (13, 18). Moreover, most BCI-FES systems reported in the literature use BCI strategies based on EEG activity related to motor-imagery or intent. However, those systems generally have limited task/command options (8, 18, 19), due to limitations of their experimental paradigms (20) and performance (21), specially with stroke (22) and other central nervous system-injured patients (23).

In contrast to endogenous, motor related BCI paradigms, other BCI modalities are more suitable for the selection of one among several targets in a variety of contexts, such as those based on steady state visual evoked potentials (24) or the P300 potential (25, 26). Moreover, these BCI systems require a short training period and can be controlled by most people, including patients with CNS injury (27). Furthermore, the nature of visual BCI paradigms make them compatible with action observation tasks, which have been reported as feasible interventions to promote motor-related brain activity (27) and enhance motor recovery in stroke patients (28).

Although there are a few reports on the use of visual BCI paradigms to control FES systems (29), most of them involve abled-bodied subjects and focus on the BCI performance, and none of them has been applied to motor rehabilitation of post-stroke patients (29). Particularly, there are no reports of a P300-based BCI controlled FES system used for upper limb rehabilitation of stroke patients. In this work we propose to explore the effects of a FEST intervention controlled by a P300-based BCI system on the recovery of ULMI in chronic post-stroke patients, assessed with a battery of relevant clinical scales.

## 2. Materials and methods

### 2.1. Patients and study design

**Figure 1:**
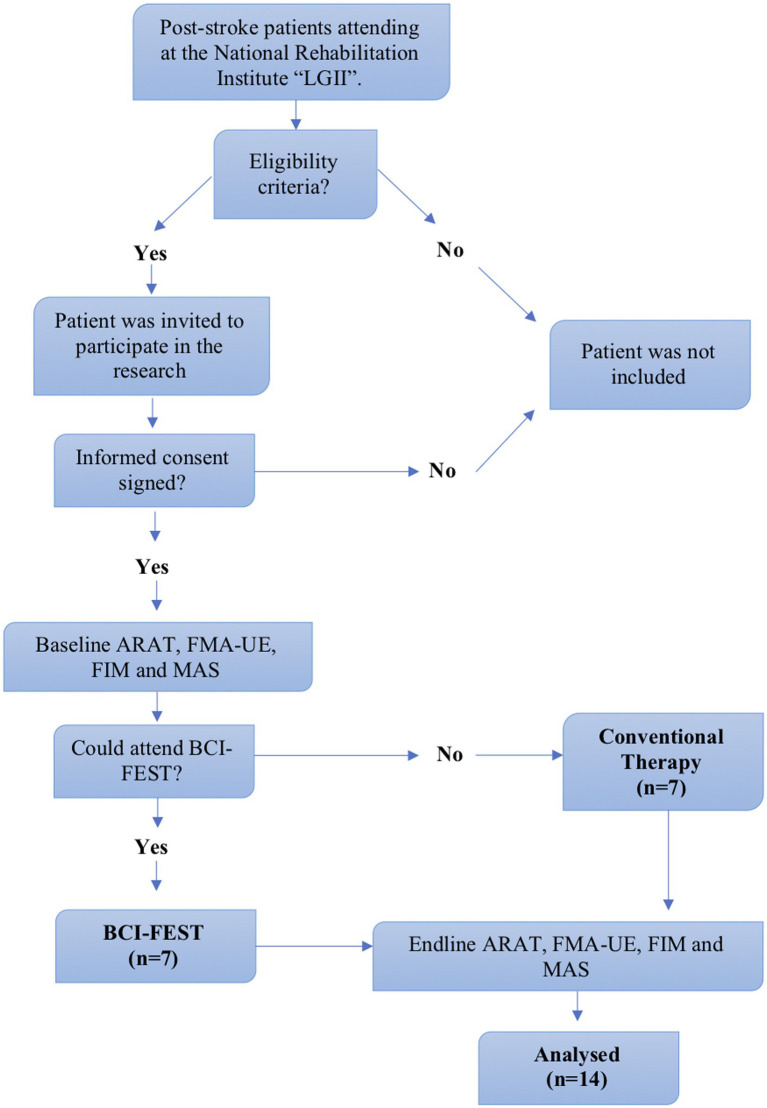
Flowchart for patient’s selection, data assessment and analysis.

A pilot study was carried out, with post-stroke individuals attending the National Institute of Rehabilitation, “Luis Guillermo Ibarra” in Mexico City. We included a convenience sample of 14 subjects divided into 2 training groups (BCI-FEST, and Conventional Therapy) by consecutive sampling. Patients that met inclusion criteria were invited to participate in the research; each participant signed a written informed consent before baseline evaluation and allocation. Four clinical scales were applied by a trained neurological rehabilitation specialist at the beginning and at the end of the intervention: Action Research Arm Test (ARAT), Fugl-Meyer Assessment Upper Extremity (FMA-UE), Functional Independence Measure (FIM) and Modified Ashworth Scale (MAS), to measure upper limb function, motor recovery, independence, and spasticity, respectively. When patients accepted to participate, and they were able to attend the training sessions with BCI-FEST, they were allocated to that group; in contrast, when patients were interested to participate but they were not able or had trouble to attend the intervention sessions in the available time schedule, Conventional Therapy was offered instead. This process was followed until completing the sample. Figure 1 shows the flowchart indicating the procedure for patient selection, follow-up and the analysis of the results after having carried out the therapy sessions.

The inclusion criteria for patients were, Adults (>18 years), diagnosed with unique stroke event confirmed by Computed Tomography (CT) or Magnetic Resonance Imaging (MRI), more than 6 months of evolution, severe hemiparesis [Fugl-Meyer Assessment Upper Extremity (FMA-UE): 0–28 points] (30), complete passive range of motion in elbow, forearm, wrist and hand; capable to keep their gaze and attention during the intervention sessions. Subjects were excluded if they had severe spasticity in hand (Modified Ashworth Scale score > 3), aphasia, epilepsy, another neurologic disease that affected upper limb function, limitations on cognitive function or metabolic conditions that limited seating. Elimination criteria included: withdrawing from research, pain, or any neurological symptoms during the training.

### 2.2. Outcome measures

Four standardized clinical scales, widely accepted for the assessment of stroke patients undergoing rehabilitation, were used to evaluate, before and at the end of the therapy sessions, upper extremity motor recovery (Fugl-Meyer: FM), upper limb function (Action Research Arm Test: ARAT), functional independence (Functional Independence Measure: FIM) and spasticity (Modified Ashworth Scale: MAS). The Fugl–Meyer (31) and ARAT (32) scales allow the assessment of different functional movements as gripping, grasping and pinching in patients with upper extremity impairment, which and are the targets of the proposed intervention since they are relevant to daily-life activities (33). The MAS scale is widely used to measure spasticity which is one of the major complications that can limit motor performance in stroke patients, particularly of the hand.

### 2.3. FEST intervention based on P300 BCI system

#### 2.3.1. P300-based BCI-FES system

Most BCI-FES systems use BCI paradigms based on EEG signals related to motor imagery or intent, to generate control signals for FES (29). Instead, in this work we propose a BCI control strategy based on a modified version of the classic P300 Donchin Speller Interface (34), where the matrix of letters and symbols is replaced by a set of pictures including five hand gestures and wrist orientations: hand opening (HO), grasping (GR), pinching (PN), pronation (PR), and supination (SP). The design of the visual stimulation interface of this BCI was previously reported by our group (29). This BCI approach is based on the oddball paradigm (35), relying on conscious recognition by the user of the intensification (change in color -blue to green- and 50% increase in size) of a particular (target) movement picture, within a sequence of other, non-target, random visual stimuli (intensification of the five pictures in a row or column that do not contain the target picture). This process should evoke the P300 component in the Event Related Potential (ERP).

**Figure 2:**
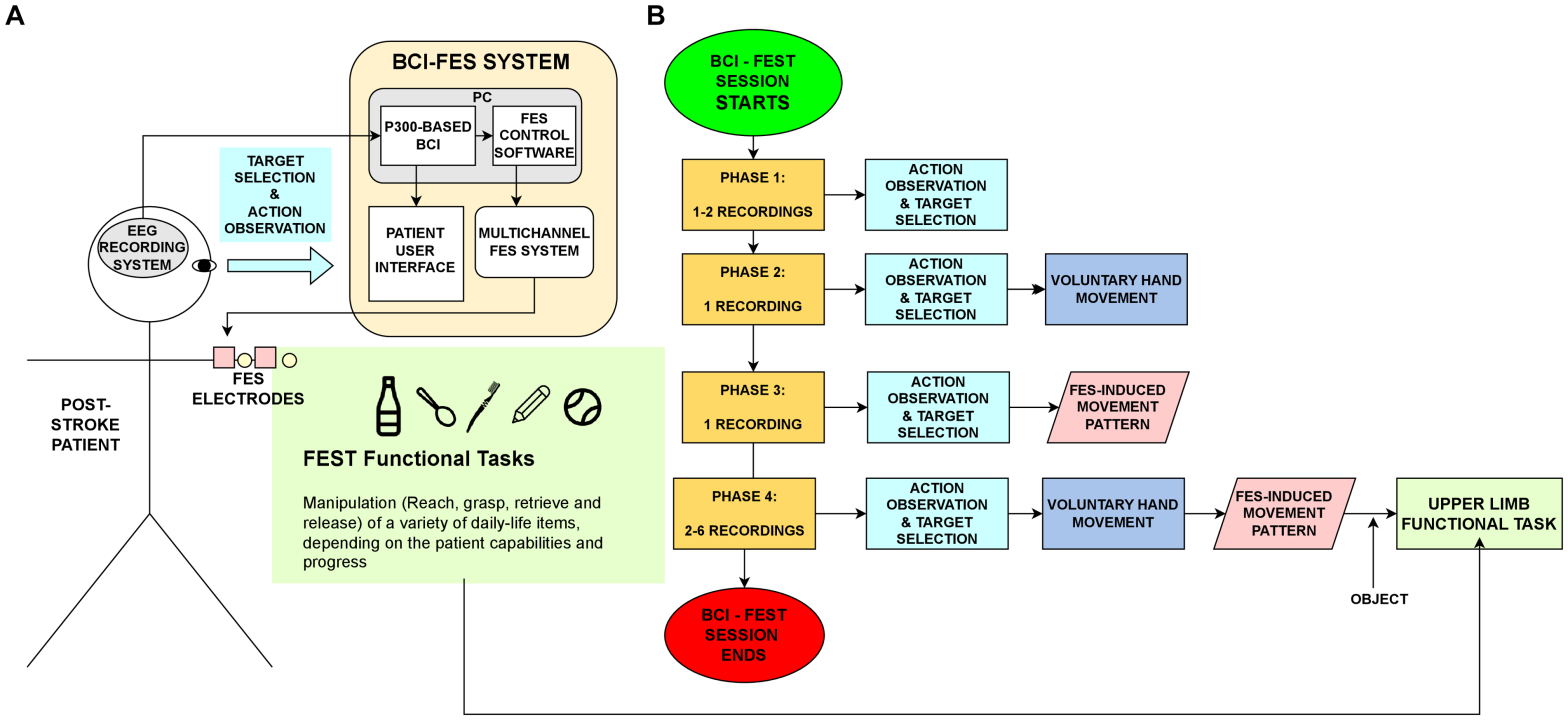
**(A)** Diagram of the proposed BCI-FEST approach. A P300-based BCI translates EEG signals related to the attended target movement in a screen, to commands that activate FES stimulation patterns. These FES patterns coordinately activate nerves and muscles in the forearm, guiding the user to perform different upper limb functional tasks. **(B)** Flow diagram of the BCI-FES sessions and the sequence of tasks performed in each of their four Phases.

A block diagram of the BCI-FES system is shown in Figure 2A. For EEG signal recording a wireless acquisition system (g.Nautilus, g.tec medical engineering, GmbH, Schieldberg, Austria) was used. EEG signals were recorded from 13 EEG channels at locations of the Extended 10–20 system (F3, F4, Cz, C3, C4, Pz, P3 P4, PO3, PO4, PO7, PO8, Oz, common reference in the right ear lobe, ground in AFz). For Electrical Stimulation the programmable electrical stimulator Rehastim 2 (HASOMED, GmbH, Magdeburg, Germany). The BCI2000 software platform (36) was used to perform all BCI tasks: EEG signal acquisition, ERP feature extraction, signal processing and classification, and finally, predicting the target movement selected by the user. Once the target movement was selected, the BCI sent the corresponding output command to the FES control block (Figure 2A), developed in MATLAB ® 2017b and Simulink ® (37), which in turn activated the FES system.

#### 2.3.2. BCI-FEST intervention

The BCI-FEST intervention consisted of the following sessions.

##### 2.3.2.1. FES calibration session

Four pairs of transcutaneous, self-adhesive stimulation electrodes (Durastick Plus, Chatanooga, DJO, LLC, Dallas, TX, USA), for four FES channels, were placed over the patient’s forearm volar and dorsal sides. Two FES channels were aimed to activate the finger/wrist flexor muscles and the other two channels to activate the finger/wrist extensor muscles. The anatomical targets of these electrodes were the median, ulnar and radial nerves, which innervate the muscles involved in wrist and fingers flexion and extension. Each stimulation channel (pair of electrodes) was directed to a particular subset of forearm muscles, described below:

- CH1 – wrist flexors (WF): flexor carpi radialis, flexor carpi ulnaris.
- CH2 – finger flexors (FF): palmaris longus, flexor digitorum superficialis, flexor digitorum profundus.
- CH3 – wrist extensors (WE): extensor carpi radialis longus, extensor carpi radialis brevis, extensor carpi ulnaris.
- CH4 – finger extensors (FE): extensor digitorum, extensor digiti minimi, extensor indicis, extensor pollicis brevis, extensor pollicis longus.
  The FES parameters for all channels were the following:
- Individual pulse shape/type: biphasic, rectangular, symmetric, constant current pulses.
- Individual pulse frequency (fixed): ~30 Hz (Interpulse interval = 33.5 ms)
- Individual pulse width: 250–450 μs, selectable in steps of 10 μs. Baseline = 300 μs.
- Amplitude envelope shape: Trapezoidal, with maximum amplitude equal to the functional threshold determined experimentally for each patient.
- Trapezoid duration: 2.5–5.0 s, selectable in steps of 0.5 s. Baseline = 2.5 s.
- Trapezoid envelope rise and fall time (fixed): 1.0 s.
- Trapezoid relative time-shift (baseline = 0.0 s):
  - Channels aimed to agonist muscles (CH1&CH2, CH3&CH4): 0, 0.5 or 1.0 s. Channels aimed to antagonist muscles (CH1&CH3, CH2&CH4): 1.5, 2 or 2.5 s.
  Using these stimulation channels and parameters, the three following FES sequences were designed to achieve a set of basic functional movements.
- Hand Opening (FS_HO):
  - CH4(FE): duration: 4.0–5.0 s.
  - CH3(WE): duration: 4.0–5.0 s, time shift to CH4 = + 0.5–1.0 s.
- Grasping (FS_GR):
  - CH1(WF): duration: 4.0–5.0 s.
  - CH2(FF): duration: 4.0–5.0 s, time shift to CH1 = + 0.5–1.0 s.
- Pinching (FS_PN):
  - CH2(FF): duration: 4.0–5.0 s.
- Hand Opening + Grasping (FS_HO + GR):
  - CH4(FE): duration: 2.5–3.0 s.
  - CH3(WE): duration: 2.5–3.0 s, time-shift to CH4 = + 0.0–0.5 s.
  - CH1(WF): duration: 4.0–5.0 s, time-shift to CH4 = 2.0 s.
  - CH2(FF): duration: 4.0–5.0 s, time shift to CH4 = + 2.0–2.5 s.
- Finger Extension + Pinching (FS_FE + PN):
  - CH2(FF): duration, 2.5–3.0 s.
  - CH4(FE): duration: 4.0–5.0 s, time-shift to CH2: + 2.0–2.5 s.

The procedure followed to configure FES parameters for each patient and stimulation channel was based on the protocol reported in (38), with additional adjustments in trapezoid duration and relative time-shift between channels. These adjustments were made at the beginning of this first FES calibration session, and later at the beginning of each BCI-FES session, to assess the current range of motion achieved through FES assistance and voluntary movements (of the shoulder and elbow) to achieve a set of target functional tasks (to be described). It is worth noting that the items used in the FES calibration session were relatively small and light (i.e., empty 250 mL water bottles) for the grasping movement, and relatively big and wide (a text marker and a soft therapy ball) for the pinching movement. Further adjustments to parameters were made at the beginning of each BCI-FES session, since bigger/smaller and heavier/lighter items were incorporated as the intervention progressed. Specifically, bigger bottles/jars filled with water or gel (up to 1 L) were used for grasping tasks and smaller and thinner objects (spoons, pencils, toothbrushes, cotton swabs, and marbles) were used for the pinching tasks.

##### 2.3.2.2. BCI calibration session

The patient was asked to visually attend two sets of 6 targets (movement pictures) on a computer screen. The order of targets in each recording of this session was: PN, SP, GR, PR, HO, and GR. While attending each individual target, 15 sequences of visual stimuli (change in background color and size of the pictures embedded in a single row or column of the matrix, at a time) were presented semi-randomly (29). EEG signals recorded in this calibration session were used to train a classification algorithm (stepwise linear discriminant analysis, SWLDA), using the P3Classifier tool of the BCI2000 platform (39). The classifier coefficients obtained in this calibration session were used during BCI-FEST session to predict the targets selected by the user.

##### 2.3.2.3. 20 BCI-FEST sessions

The aim of these sessions was to link an action observation/target selection task, mediated by the P300-based BCI, with the practice of an FES-assisted functional task involving the target movement picture selected. The functional tasks involved manipulation of common objects in daily-life activities (bottle, pencil). Each BCI-FEST session lasted 1 h/day, 2–3 days/week until completing 20 sessions. Patients in this group also received conventional physical therapy but not occupational therapy, since the latter involves more specific therapy for the upper limb. BCI-FEST sessions consisted of a sequence of recordings (shown in Figure 2B) comprising four phases, which were progressively integrated during the session:

- Phase 1 recordings (1–2): Action observation & target selection. The user was asked to attend (during 20–30 s) only one specific target movement picture, and to mentally count the number of visual intensifications of the target detected consciously. They were instructed to observe the target picture while thinking as if it was his/her own hand performing the attended gesture during the visual intensification period. In these recordings the user received feedback of the BCI predicted target and served as a baseline to identify and correct technical or human issues. The order of targets in this phase was PN-SP-GR-PR-HO-GP.
- Phase 2 recordings (1): Voluntary movement. Besides the indications given in phase 1, the patient is asked to perform voluntarily, or at least try to perform the hand gesture he or she was attending when using the BCI interface, as soon as the sequence of visual stimuli corresponding to the current target is over. No FES feedback is still given at this phase. The order of targets in these recordings was: PN-GR-HO-PN-GR-HO.
- Phase 3 recordings (1): Passive FES movement. The user is asked to perform the Phase 1 Task and when receiving the FES feedback, just observe the movement induced by FES alone, avoiding performing any voluntary movement. For this, the BCI system outputs a command to the FES control system (Figure 2A), informing the predicted user target. Then, the FES control system executes a multichannel FES pattern that induces the target hand gesture, while the patient keeps the elbow flexed at approximately 90° and neutral wrist orientation. The order of targets in these recordings was the same as those in Phase 2: PN-GR-HO-PN-GR-HO. In this phase and the next, the following FES sequences described in the FES calibration section were sent whenever the movement commands (pictures) were selected through the BCI system: FS_FE + FF fo the PN command, FS_HO for the HO command, FS_HO + PG, for PG command.
- Phase 4 (2–6 recordings): Functional task. In addition to the Phase 3 tasks, the user is instructed to perform a functional task involving the manipulation (reaching to, hand opening, grasping, moving/retrieving, and releasing) of some common daily life item, by combining the voluntary movement with the FES induced movements. Similarly to Phase 3 recordings, the FES patterns in Phase 4 were activated once the BCI predicts the user selected targets, which were six consecutive pictures of the same type (6xGP, 6xHO or 6xPN) or alternated between HO and GP or PN (HO-GP-HO-GR-HO-GR or HO-PN-HP-PN-HP-PN). The main difference here is that users are instructed to synchronize their voluntary movements with the ones induced by FES, to achieve the functional target goal. This way, the FES pattern serves as a time specific guide to perform the target functional movement, which involves the target movement previously observed/selected in the P30-based BCI. Some objects employed in this Phase included: bottles, pencils, spoons, cotton swabs, pellets. A typical functional task consisted in: (1) reaching for the object, (2) opening the hand, (3) grasping the object, (4) retrieving/moving the object to a useful position, (5) opening the hand to release the object. Session by session, the type, size and weight of the items were adjusted, according to the capabilities of the patient, which in general were enhanced throughout the intervention. The FES patterns obtained in the basal FES calibration session, were adjusted for each patient at the beginning of each BCI-FES session to make the movements more comfortable, reduce fatigue or non-selective activation of muscles, increase range of motion and grasping/pinching force (adjusting amplitude or pulse width), and in general to make the movements more functional and natural. This was achieved by adjusting the width of the individual stimulation pulses (300–500 μs), and the maximum amplitude (4–30 mA), duration (0.5–5 s) and relative time-shift (0.5–2.5 s) of the trapezoidal FES patterns for each of the four FES channels.

For performance assessment of the use of the BCI-FES system for each patient, the percentage of correctly selected (PC) commands over the total of target selection tasks in a BCI session was calculated as follows:

(1) $$PC=\frac{CSC}{TST}\times 100$$

where CSC is the number of correctly selected targets, and TST is the total number of selection tasks.

In other words, this parameter indicates (in percentage) the number of times that the BCI correctly predicted the specific movement picture selected (attended) by the patient in the user interface, against the total number of command selection tasks for recording (generally 6 tasks for recording).

### 2.4. Conventional therapy

Conventional therapy was based on 20 sessions of joint mobility, muscle strength, task-specific training, sensitivity reeducation and coordination exercises (physical and occupational therapy) directed by an experienced professional therapist. These sessions lasted 1 h daily, 5 days/week for 4 weeks.

### 2.5. Statistical analysis

Statistical analysis was performed using the software Statistical Package for the Social Sciences (SPSS® v25.0, IBM®) and GraphPad. First, the normality of the demographic data and the outcome measures was evaluated using the Kolgomorov Smirnov test (*α* = 0.05), to determine if parametric or not parametric statistics would be used. Then, descriptive analysis was realized for the sociodemographic and clinical outcome variables. The between-group differences of clinical characteristics in baseline were evaluated through the Chi-square test for categorical variables (MAS) and for numerical variables (FMA, ARAT, and FIM) through the two-sample *t*-test or the Mann–Whitney *U* test depending on the results of the normality test. The same tests were used for the comparison of quantitative clinical outcome measures (FMA, ARAT y FIM) before and after the training, gains in quantitative clinical variables (FMA, ARAT y FIM) and the comparison between groups of spasticity assessment (MAS).

For all tests the statistical significance level was set at *α* = 0.05. Bonferroni corrections were applied in the comparison of quantitative clinical outcomes, before and after training, to adjust probability, and avoid risk of a type I error. The effect size was calculated with Cohen’s D for the difference between groups (comparisons with Mann Whitney *U* test) on the G*Power software. Finally, the required sample size for a randomized controlled trial was calculated with the software EpiData v6.

## 3. Results

Fourteen individuals with hemiparesis secondary to stroke were included in the study. All patients in both groups completed the 20 scheduled sessions. As for the BCI-FEST group, one patient had to reschedule 2 sessions 1 week later because of ambulatory ophthalmologic surgery that did not affect patient participation subsequently. Another patient had to reschedule 2 sessions because he reported the stimulation to be uncomfortable twice. After three rest days recommended by the physician in charge, BCI-FEST sessions were restarted without another symptom until the end of the sessions. No patient withdrew their consent during the research and no adverse event related to the intervention occurred.

Most patients were male (57.1%) with a mean age of 52 years (SD: 20.2 years, range: 18–79 years). In terms of stroke, the majority had an ischemic etiology (64.3%), with an average evolution time of 19.9 months (SD: 24.4). In the comparison between groups (Table 1) no differences were found in terms of sex, etiology, and evolution time.

**Table 1:** Sociodemographic and clinical characteristics of patients.

Variable	Frequencies/mean	BCI-FES	Conventional therapy	*p*-value
Sex				
-Men	8 (57.1%)	5 (71.4%)	3 (42.8%)	0.280ª
-Women	6 (42.9%)	2 (28.6%)	4 (57.1%)	
Stroke etiology				
-Ischemic	9 (64.3%)	4 (57.1%)	5 (71.4%)	0.801^a^
-Hemorrhagic	3 (21.4%)	2 (28.5%)	1 (14.2%)	
-Both	2 (14.3%)	1 (14.2%)	1 (14.2%)	
Age (years)	52 (SD 20.2)	51.8 (SD 20.3)	54.1 (SD 21.4)	0.906^b^
Time since injury (months)	19.9 (SD 24.0)	27 (SD 33.6)	12.8 (SD 6.3)	0.057^b^

^a^Chi square, ^b^Mann–Whitney *U*.

From the normality test, it was found that not all variables were normally distributed, then the non-parametric test (Mann–Whitney *U* test) was used for comparisons on the Fugl-Meyer, ARAT, and FIM variables.

Table 2 shows the comparison between groups of quantitative clinical outcomes measures, before and after training. At baseline, no difference between groups existed on the Fugl–Meyer and FIM variables. After training, we found statistically significant differences between groups for the Fugl–Meyer, the ARAT and the FIM scales, as is shown on Table 2. The improvements were greater in the BCI-FEST group for the Fugl–Meyer and the FIM scales in the comparison between groups of gains in quantitative clinical variables. We did not find statistically significant differences in initial MAS (*p* = 0.192) and the final MAS (*p* = 0.485) between groups.

**Table 2:** Between-group comparison of quantitative clinical outcome measures, before and after the training.

Variable	Baseline Mean (SD)	*p*-value	Final Mean (SD)	*p*-value	Gain mean (SD)	*p*-value
Fugl-Meyer -BCI-FES -Conventional Therapy	26.1 (11.5) 10.5 (5.2)	0.236^b^	38.7 (16.5) 14.29 (4.3)	0.012^b^	12.5(10.5) 4.7 (3.4)	0.024 ^b^
ARAT -BCI-FES -Conventional Therapy	16.7 (11.5) 2.2 (1.7)	0.006 ^b^	26.7 (18.3) 4.8 (6.3)	<0.001^b^	10 (9.7) 3.5 (4.9)	0.198 ^b^
FIM -BCI-FES -Conventional Therapy	102.8 (11.2) 61.29 (33.5)	0.068 ^b^	115.2 (33.5) 66.4 (33.2)	0.025^b^	1.24 (12.5) 2.5 (2.7)	0.028^b^

^b^Mann–Whitney *U*.

From the results obtained in this work, a high effect size was obtained for the Fugl-Meyer (0.99) and ARAT (0.84) scales, and a low size effect value for the FIM scale (0.13). Finally, from the sample size calculation, it was determined that 28 patients (14 for each group) are required in order to detect a difference between means of 7.8 (SD: 7.1) in the Fugl–Meyer UE scale, a power of 80% and a confidence level of 95%. Regarding the performance of the BCI system, a mean (S.D.) PC of 58.66 (28.03) was obtained for all patients. For each patient the mean (S.D.) PC is reported below:

- P1: 87.83 (41.90)
- P2: 44.34 (21.75)
- P3: 83.15 (12.64)
- P4: 65.16 (19.31)
- P5: 22.50 (13.10)
- P6: 25.00 (11.78)
- P7: 82.63 (16.69)

## 4. Discussion

Our results indicate that there was greater improvement in upper limb function, motor recovery, and functional independence in the BCI-FEST group compared to the Conventional Treatment group. These results are in alignment with previous reports of ULMI improvements in stroke individuals using BCI-FES systems, which could represent changes due to neuroplasticity mechanisms (40, 41). Neuroplasticity refers to all structural and functional dynamic changes in the brain associated with learning, memory, adaptation, and rehabilitation, and it is influenced by many neural processes that depend on expressions of genes and epigenetic mechanisms (42). It is very well described that the period of greatest spontaneous motor recovery is within the first 3–6 months after stroke and that rehabilitation strategies used in the recovery process influence this spontaneous neuroplasticity (43). However, recent studies have proposed a wider interval of time than the commonly accepted for the recovery of ULMI through technology-based therapies. For example, Ballester et al. (44) demonstrated significant improvement in Fugl–Meyer score in patients up to 18 months after stroke with occupational and virtual reality therapies (15). On the other hand, Remsik et al. reported meaningful functional improvements after 1 month of BCI sessions performed within the first 3 years after stroke (18).

According to the scientific literature, BCI-FES possess the ability to induce neural plasticity by allowing real time feedback of neural activity synchronizing facilitated motion with detected brain activity during the functional task practice (18, 45). However, it should be noted that the FES control approach proposed in this work (P300-based BCI system with visual paradigm) differs from the ones used by the above mentioned studies, which are based on electromyography signals (46–48), electroencephalography signals with a visual cursor feedback (18), or motor imagery *via* mu-rhythm desynchronization (49). In fact, most BCI-FES systems for applications to motor rehabilitation are based on motor imagery/intent strategies, and the associated changes in sensorimotor EEG rhythms (29). However, those systems have limitations in performance (23), and the number of selectable targets (19), that restrict the functional tasks to be performed in BCI-FEST sessions.

For this study, we designed an alternative BCI-FEST control strategy that allowed us to present up to five different commands, in the form of upper limb movements pictures. At the same time, the BCI system required the user to engage in an action observation task, which was necessary for the system to determine the user selected command. Action observation has been used to promote motor-related brain activity (27) and enhance motor recovery in stroke patients (28, 50). Since action observation is known to activate the motor mirror system, to enhance motor recovery and to induce plasticity in post-stroke patients (51), we suggest that the results obtained in this study can be explained, at least in part, by the proposed BCI-FEST approach. Moreover, this BCI-FEST is fully aligned with the therapeutic approach proposed by Ertelt et al. (51): combining action observation with actual performance of the action observed.

Interestingly, our results show that patients in the BCI-FEST rehabilitation therapy group, despite having a longer evolution time (27 ± 33.6 months) than conventional therapy group (12.8 ± 6.3 months), had better, clinically significant, changes in upper limb motor recovery with a difference of 12.5 points in FMA-UE compared with conventional therapy group (4.2–7.2 FMA-UE) (52). The study of (41) showed similar differences between groups after BCI training for 4 weeks but the differences in FMA-UE were not clinically significant. In the same year, Sebastián-Romagosa et al. showed that BCI therapy improved FMA-UE and spasticity with clinical significance after 25 sessions. It is important to highlight that the subjects in those studies had a shorter post-stroke evolution time than ours, since we included patients up to 7 years post-stroke obtaining significant improvements in ULMI. This degree of motor recovery in chronic stroke individuals is not commonly seen and challenges the known paradigms of neuroplasticity after stroke (15, 46, 53, 54). However, we still need more evidence of the phenomenon, as with a task-based functional magnetic resonance, to support this theory about changes in neuroplasticity after the intervention.

One of the most clinically relevant outcome measures in post-stroke subjects is upper limb functionality, since it is related to performance of activities of daily living, including self-care and social activities (31, 32, 55). Some studies have shown statistical differences in ARAT scores between BCI-FEST and conventional therapy but without clinical significance (47–49). We found statistically significant differences in the ARAT score after intervention in the BCI-FEST group. When comparing the gains on both groups, we found that they are similar, although the difference was clinically significant only for the BCI-FEST group. This may be explained in part due to the initial score of upper extremity function being higher in the intervention group. Regarding the FIM, both groups had improvements in the score, but they were not clinically significant (33). Finally, there were no differences between groups in spasticity before and after training; nevertheless, the BCI-FEST group tended to show a decrease in this variable. Considering that this group was the one that achieved the greatest motor recovery, these changes could follow the line based on Twitchell and Brunnstrom’s concept of sequential stages of motor return in hemiplegic stroke patients where it is expected that to greater motor recovery, the lesser the spasticity (52, 56–58). Although the sample size of the study is small, we found a large effect size in the numerical clinical variables, indicating a likely advantage of the P300-based BCI-FEST for ULMI recovery, when compared with the conventional therapy.

It is worthwhile to mention that most patients in the BIC-FEST group reported positive perceived benefits during and after their participation in the study, in particular increased independence and participation in diverse activities involving the use of their affected hand in functional contexts. Examples of those reported activities include taking a bath alone, helping in the family business preparing and delivering food, being part of a competitive team of wheelchair basketball, improved ability to perform bimanual tasks in their economic activities and daily life etc. More importantly, they reported increased motivation to further improve their hand function, continue their professional/technical studies, to drive a car again, to get a job where they use both hands.

No adverse events related to the intervention occurred, the only situation reported by one patient was that in two consecutive sessions he reported high sensitivity to electrical stimulation, even below the motor threshold of previous sessions. The patient was examined by a neurologic rehabilitation specialist (ARN), who recommended 3 days rest, later the intervention sessions were resumed, taking extra care to re-calibrate the stimulation electrode placement and parameters, and there were no further issues from then.

Finally, FES calibration (electrode positions and parameters) was revised at the beginning of each BCI-FEST session, and slight adjustments were made as required, i. e. when the expected movements were not reached in terms of range of movement or force (to hold the objects to be manipulated in the session), or if they were uncomfortable for the patient (generally if a score of 6 out of 10 or greater in the Wong-Baker Faces scale was reported). In general, the stimulation amplitude needed to achieve functional movements was reduced throughout the sessions.

A few times, the expected FES patterns were not delivered due to bad connection in electrode leads or defective electrodes, or failure in the communication between the control software and the Rehastim 2 stimulation device. These issues were resolved by reconnecting firmly the stimulation cables to the electrodes or changing the electrodes, or resetting the computer and the stimulator and the USB connection between them.

## 5. Conclusion

In the present pilot-study, a FEST intervention for ULMI rehabilitation, using a P300-based BCI system as control strategy, was tested in a sample of stroke patients. These first results show that this modality of BCI- FEST was more efficient than conventional therapy to improve ULMI after stroke, regardless of chronicity. Therefore, the proposed intervention is promising for the application of a safe and non-invasive technology for rehabilitation of ULMI post-stroke, even in the most chronic patients, who are generally relegated from novel interventions, and their expected recovery with conventional therapy is very low. It is necessary to carry out a randomized controlled trial in the future with a larger sample of patients, where this BCI-FEST approach is compared with conventional therapy and possibly other control modalities of BCI-FEST or even FEST alone.

## 6. Limitations of the study

As mentioned in section 2.1, some patients that were interested in participating in the BCI-FEST intervention but were not able to attend the scheduled sessions, were allocated to the Conventional Therapy group. The frequency of the BCI-FEST intervention sessions was established according to the available time schedule of the researcher in charge of the sessions, which was not always compatible with the possibilities of patients and caregivers, due to logistical reasons. The limited time schedule for BCI-FEST sessions was due in part to additional time needed for various tasks performed by the experimenter before and after the sessions (preparation and maintenance of equipment, software, electrodes, documentation, data processing, etc.).

For the Conventional Therapy group, full-time rehabilitation personnel and logistics were available to attend patients, as part of their daily job schedule. In particular, Conventional Therapy was available early in the morning, which was more convenient for the patients and caregivers. Some of these differences could have affected potential benefits to patients in the BCI-FEST, if they had received therapy sessions more often.

On the other hand, patients allocated in the Conventional Therapy group, knowing they were not included in the novel therapy group, could have been affected in their motivation, engagement or confidence in the potential benefits of the conventional physical and occupational therapy, or in their efforts in the clinical assessment sessions. To avoid these issues, in future trials, logistical and personnel requirements will be taken fully into consideration in order to provide similar intensity to patients in both the BCI-FEST and the Conventional groups. Also, a more intensive patient recruiting campaign will be performed, in order to have more patients from the beginning of the trial, and to facilitate random allocation and blinding of clinical evaluations.

Another limitation of the study is that we have not applied any standardized assessment related to patient participation, which could have given us clinically relevant information. In the next stage of the project we will evaluate this aspect through a general health questionnaire and the quality-of-life questionnaire.

Regarding possible sources of bias, we identify two of them. The first one is lack of blinding during the process of patient assignment to BCI-FEST or conventional therapy groups, which was not possible due to the design of the study and the limited number of patients who complied with the selection criteria. The second likely source of bias is the lack of blinding of the medical specialist who performed the clinical assessments, regarding group allocation of patients. Although the specialist is highly trained in the application of these assessments, and she was focused on using with all patients the same indications and scoring criteria, we cannot discard some bias toward the BCI-FEST group. This issue can be minimized in a future, randomized, blinded study, with a higher sample size.

An additional likely source of bias is the ARAT value at baseline, which was lower for the Conventional Therapy group. This could have happened due to the lack of randomization of patient allocation. However, at the end of the therapy sessions, no difference between groups were found in gains in this scale. In contrast, for Fugl–Meyer and FIM, which had no differences at baseline, there were statistically significant differences in gains at the end of the intervention, being in both cases higher for the BCI-FEST group. For a future randomized controlled study, randomization will be used which will likely help to prevent this.

## Data availability statement

The data that support the findings of this study are available from the corresponding author, upon reasonable request. Requests to access the datasets should be directed to josefina_gutierrez@hotmail.com.

## Ethics statement

The research was conducted in accordance with the Declaration of Helsinki and was approved by the Research and Ethical Committees of the National Institute of Rehabilitation “Luis Guillermo Ibarra Ibarra” (Registry number 17/21). The studies were conducted in accordance with the local legislation and institutional requirements. The participants provided their written informed consent to participate in this study.

## Author contributions

JG-M: conceptualization, supervision, writing, review, and editing. AR-N: conceptualization, supervision, performed the research, analysis, writing, review, and editing. JQ-F: conceptualization, supervision, analysis, writing, review, and editing. JM-G: performed the research, analysis, writing, review, and editing. CT-P, GV-M, and MG: data curation, writing, and editing. MP-G: supervision, writing, and editing. CH-A: writing, review, and editing. All authors have approved the final version of the manuscript.

## Funding

This work was supported by the Secretaría de Educación, Ciencia, Tecnología e Innovación (SECTEI) de la Ciudad de México, through grant SECTEI/183/2019, and by the Consejo Nacional de Humanidades, Ciencias y Tecnologías de México (CONAHCYT), through grant FOP16-2021-01-319246.

## Conflict of interest

The authors declare that the research was conducted in the absence of any commercial or financial relationships that could be construed as a potential conflict of interest.

## Publisher’s note

All claims expressed in this article are solely those of the authors and do not necessarily represent those of their affiliated organizations, or those of the publisher, the editors and the reviewers. Any product that may be evaluated in this article, or claim that may be made by its manufacturer, is not guaranteed or endorsed by the publisher.
